# Hemodialysis Patients’ Emotional Profiles and Associated Symptomatology: A Cross-Sectional Multicenter Study

**DOI:** 10.3390/nursrep15050152

**Published:** 2025-04-30

**Authors:** Ana Casaux-Huertas, Pilar Mori Vara, Maria del Carmen Hernández-Cediel, David Hernán-Gascueña, Rosa M. Cárdaba-García, Veronica Velasco-Gonzalez, Lucía Pérez-Pérez, Miguel Madrigal, Inmaculada Pérez, Carlos Durantez-Fernández

**Affiliations:** 1 Fundación Renal Española, C/ José Abascal 42, 28003 Madrid, Spain; 2 Department of Nursing, Faculty of Nursing, Physiotherapy and Podiatry, Complutense University of Madrid, Pl. de Ramón y Cajal 3, 28040 Madrid, Spain; 3 Fundación Jiménez Díaz School of Nursing, Autonomous University of Madrid—Instituto de Investigación Sanitaria FJD, Av. de los Reyes Católicos 2, 28040 Madrid, Spain; 4 Department of Nursing, Faculty of Nursing, University of Valladolid, Av. Ramón y Cajal 7, 47005 Valladolid, Spain; 5 Nursing Care Research (GICE), Faculty of Nursing, University of Valladolid, Av. Ramón y Cajal 7, 47005 Valladolid, Spain; 6 Primary Care Management Valladolid West (SACYL), C/Dulzaina 2, 47012 Valladolid, Spain; 7 IOBA (Institute of Applied Ophtalmobiology), University of Valladolid, Pº Belén 17, 47011 Valladolid, Spain

**Keywords:** renal dialysis, kidney failure, chronic, emotions, signs and symptoms, depression, nursing, cross-sectional studies

## Abstract

**Background**: Chronic kidney disease (CKD) has a significant impact on patients’ physical, psychological, and social well-being. Emotional disorders are common and contribute to a higher prevalence of symptoms compared to that in the general population. This study aimed to analyze the relationship between the emotional profiles and symptomatology in patients undergoing hemodialysis (HD). **Methods**: A multicenter, cross-sectional, observational/analytical study was developed in seven centers of the Spanish Renal Foundation in the Community of Madrid (Spain). The study protocol was reviewed and approved by the Clinical Research Ethics Committee of Hospital Clínico San Carlos, Madrid (C.I. 20/685-E). In the study, two validated measurement scales were used: the Mood Rating Scale (EVEA) to assess the “emotional profile” and the Palliative care Outcome Scale, Renal Symptoms (POS-S Renal) to evaluate “symptomatology”. **Results**: The sample (245 patients) was predominantly male (65.7%; n = 161), with a mean age of 63.52 years (SD = 14.99) and an average HD treatment duration of 81.44 months (SD = 96.62). The analysis of the symptom–emotion relationships revealed that patients with a sadness–depression profile had a higher probability of experiencing weakness or a lack of energy (OR = 1.741; CI 95% 1.01–3.00) and feelings of depression (OR = 3.236; CI 95% 1.98–5.30). Additionally, patients with an anger–hostility profile exhibited a significant association with pain (OR = 3.463; CI 95% 1.34–8.94) and excessive sleepiness (OR = 3.796; CI 95% 1.21–11.95), indicating that this emotional state substantially increases the likelihood of developing these symptoms. **Conclusions**: The emotional profiles of CKD patients undergoing HD significantly influence their symptomatology. While positive emotions may play a protective role in preventing debilitating symptoms, negative emotions increase the risk of their onset. These findings highlight the importance of addressing emotional well-being as part of comprehensive care for HD patients.

## 1. Introduction

Despite the advances made in recent decades in the treatment of chronic kidney disease (CKD), it continues to cause significant problems and changes, not only physically but also psychologically and socially, in patients who receive it, as it produces an alteration in the activities of daily life of the person undergoing treatment, which greatly affects their adherence to treatment and coping with the disease [1]. Recent studies estimate that approximately 700 million individuals worldwide are affected by CKD, with the prevalence increasing by 33% between 1990 and 2017. Notably, the highest growth in the CKD burden is observed outside high-income countries, particularly in nations like India and China [2,3]. Furthermore, projections indicate that the number of individuals requiring kidney replacement therapy could rise from 2.6 million in 2010 to 5.4 million by 2030 [2,3]. These data reflect not only the growing magnitude of the disease but also the urgent need to address its impact from a comprehensive, multidimensional perspective. It should be noted that in addition to all of its symptoms and organic discomfort, there are the limitations to which patients are subjected (those inherent to the treatment, dietary restrictions, etc.) and conditioning that attending hemodialysis (HD) sessions entails in their daily lives. In addition to the sociodemographic and clinical variables that influence patients’ perceptions of their quality of life, emotional factors must be added, namely, according to various studies [4,5], anxious, depressive, or stressful symptoms. People with chronic diseases, including patients with CKD, are more likely to suffer mood disorders due to the mere fact of having the disease since it alters their daily life and therefore negatively alters their emotional state [6]. The prevalence of depressive disorders in the population with CKD undergoing HD ranges between 25.8% [7] and 68.1% [8], while anxious disorders prevail between 21% [9] and 35.3% [10] of the population undergoing HD-type renal replacement therapy, with both emotional states having an inversely proportional influence on the quality of life of the people who suffer from them [5,11]. Likewise, these negative emotions will influence the appearance and manifestation of their symptoms. Thus, stress states and negative emotions, such as anxiety and depression, can accentuate and aggravate the clinical course of the disease, interfere with treatment, and increase the associated symptomatology, significantly increasing morbidity and mortality and acting as a predictor of survival [1,12,13]. Nurses, due to the close relationships with CKD patients, are essential in identifying and addressing negative emotional states early. Their proactive intervention can mitigate the impact of anxiety and depression, preventing the exacerbation of symptoms and improving clinical outcomes [14,15,16].

There is evidence that the presence of health symptoms in HD patients is significantly higher than that in the healthy population, and these symptoms are related to the presence of negative emotions such as anxiety and depression, the former being the most conditioning when it comes to the development and intensity of symptomatology [4,12,17]. On the other hand, positive emotions such as joy or optimism have been shown to have protective effects on delaying the onset of symptoms and even improving the course and prognosis of the disease [18]. Nurses are in a privileged position to promote positive emotional states through their continuous support, education, and therapeutic communication. By fostering emotions such as joy and optimism, they can contribute to alleviating symptoms, improving patients’ well-being, and reinforcing the vital impact of emotional care.

Despite the evidence regarding the emotional distress experienced by patients undergoing HD, the current research has mainly focused on isolated variables, such as anxiety or depression levels, rather than exploring the broader emotional profiles that may influence the development of physical symptoms. There is a lack of studies that analyze the interplay between both positive and negative emotional states and the symptomatology reported by patients with CKD undergoing HD. This gap in the literature limits the design of holistic and effective care strategies that address both the psychological and physical dimensions of a patient’s experience. Understanding these relationships could lead to more tailored and impactful interventions, especially from nursing professionals, who play a key role in the day-to-day emotional and physical care of these individuals.

Therefore, this research is based on the hypothesis that the emotional profile of patients with CKD on HD plays a determining role in the development of their physical symptoms. Hence, the objective of this research was to establish the relationship between the emotional profiles and the symptomatology developed in patients undergoing HD treatment, which will allow us to have a greater field of knowledge, thus favoring the best possible care and guaranteeing the physical and mental health of these patients through the interventions of a multidisciplinary team, where nursing professionals, as a cornerstone of care, contribute decisively to symptom monitoring and early emotional detection, ensuring comprehensive and high-quality care.

## 2. Materials and Methods

### 2.1. The Study Design

This multicenter, cross-sectional, observational/analytical study was conducted according to the “Strengthening the Reporting of Observational studies in Epidemiology” (STROBE) statement [19].

### 2.2. The Setting

This study was conducted in 7 out of the 8 centers of the Spanish Renal Foundation of the Community of Madrid. Although initially all centers were invited to participate, the “Los Llanos” Center (Móstoles) ultimately declined and thus was not included in the final sample. The participating centers were the “Santa Engracia” Center (Madrid), “Los Llanos II” Center (Getafe), “Los Lauros” Center (Majadahonda), Fundación Jiménez Díaz University Hospital (Madrid), Rey Juan Carlos University Hospital (Móstoles), Infanta Elena University Hospital (Valdemoro), and Villalba General University Hospital (Villalba).

The data collection took place from the beginning of February 2021 to the end of May 2021. After this period of time, the database was constructed, and an in-depth analysis of the results obtained was started. In order to centralize the data collection, two questionnaires were sent from the headquarters of the Spanish Renal Foundation to the eight participating centers in the Community of Madrid, accompanied by an information sheet addressed to the nursing staff. This document included detailed instructions on the correct completion of the questionnaires, as well as the criteria for the inclusion and exclusion of the participants.

The nursing staff at each center was responsible for the distribution and subsequent collection of the questionnaires, which were sent to the central headquarters for filtering and analysis by the principal investigator.

### 2.3. The Participants

Patients over 18 years of age diagnosed with advanced-stage 4–5 CKD who had been on HD treatment for at least 3 months were included in this study and had previously been informed of the purpose and aim of the project and agreed to participate in the study by signing the informed consent form. Patients diagnosed with a psychiatric pathology or cognitive impairment or who had language barriers that prevented the correct completion of the questionnaires were excluded from the sample.

### 2.4. The Study Size

To determine the sample size, we estimated the population proportions [20] based on the population size in 2020 across the centers included in the study (662 patients). A 95% confidence level and a 5% margin of error were applied, resulting in a required sample size of 243 participants.

### 2.5. Variables

The main variable determined was the study of the patients’ emotional profile, and this construct was quantified through the measurement instrument “Mood Rating Scale” (EVEA). The EVEA, created and validated in the Spanish population by Jesús Sanz [21], assesses the transient emotional states of anxiety, depression, hostility, and happiness with high content validity (an α coefficient between 0.88 and 0.93), test–retest reliability (correlations between 0.55 and 0.88), and factorial and discriminant validity for the subscales and positive and negative mood states. The EVEA scale is a tool that consists of a Likert-type scale where a score is obtained for each of the items between 0 and 10. The results obtained in the different items are grouped into four subgroups, according to the rules of use of the scale [22], which make up the emotional profiles that will be used when interpreting the results: anxiety, anger–hostility, sadness–depression, and happiness. Each emotional profile scores between 0 and 40 points. A higher score on the EVEA subscales indicates, respectively, a higher level of anxiety, anger–hostility, sadness–depression, or happiness in the person being evaluated [23].

As for the determinant variable of the associated symptomatology, the second main variable in this study, this was obtained using the self-administered Likert-type tool “Palliative Care Outcome Scale-Symptoms Renal” (POS-S Renal), translated and validated in Spanish [24,25]. The POS-S Renal was created by the “Palliative Care Outcome Scale” development team, composed of Professor Irene J. Higginson et al. The Spanish version of this instrument was adapted and validated by Daniel Gutiérrez Sánchez et al. and presents a high content validity index (an α coefficient = 0.91), test–retest reliability of r = 0.89, and internal consistency α = 0.78, which indicates that it is an instrument that reliably reflects the most prevalent symptoms in patients with CKD [24,25]. Each of the 19 different symptoms included in the scale is given a score between 0 and 4, with scores equal to or greater than 2 being considered clinically relevant because they have a negative and limiting impact on a patient’s daily life from this point onwards [25]. The interpretation of the scores according to the POS-R Renal scale is as follows: 0 points: none, no effect; 1 point: slightly, but not enough to treat; 2 points: moderately, limits some activities or concentration; 3 points: strongly, activities or concentration is noticeably affected; and 4 points: unbearable, unable to think of anything else. This was transferred to the statistical analysis as a dichotomized variable according to the presence or absence of limiting symptoms for the patient, considering scores of 0 and 1 as not limiting and symptoms with ≥2 points as limiting for the sufferer. It is important to note that the POS-S Renal scale does not yield a total or global score. Each item (symptom) is assessed and interpreted individually, allowing for a symptom-by-symptom analysis rather than a cumulative evaluation. This approach facilitates a more precise understanding of which specific symptoms are most distressing for each patient.

The secondary variables in this study were as follows:

- Sociodemographic variables: Age, sex, and center where treatment is received. These variables were obtained from the Nefrosoft software, version 7.0.1 (Visual-limes, Valencia, Spain).
- Clinical variables: Etiology of kidney disease, time on treatment, dialysis dose (Kt), interdialytic weight gain, vascular access type, and Charlson Comorbidity Index adjusted for renal disease (CCI). These variables were obtained from the Nefrosoft software and represent the average values recorded during the six months prior to the administration of the survey.

### 2.6. The Data Collection and Information Sources

The information analyzed was collected from the clinical history of each of the patients who participated in this study and from the two measurement instruments provided to these same patients by the nursing staff of the collaborating hospitals.

### 2.7. The Statistical Analysis

A descriptive analysis of the variables was performed, summarizing qualitative variables as percentages and frequencies and quantitative variables as the mean, standard deviation (SD), and 95% confidence interval (CI 95%). The Kolmogorov–Smirnov test was used to check the normality of the data, and parametric and nonparametric tests were used as appropriate. In order to evaluate the relationship between symptoms and emotional profiles, a multivariate logistic regression analysis was conducted for each symptom, adjusting for baseline covariates, including age, sex, time on dialysis (months), average final Kt, and interdialytic weight gain, given the documented confounding effects of these variables on the manifestation of certain symptoms within the study population. Initially, univariate regression models were applied to each symptom and emotional profile. Only associations with a *p*-value < 0.30 were included in the subsequent multivariate analysis. Then, a backward selection procedure was used to refine the models and control for potential confounding factors. The goodness-of-fit of the models was assessed using the Hosmer–Lemeshow test, with models having a *p*-value > 0.1 considered to have a good fit. Finally, the patients were classified based on the emotional profile for which they had the highest score. A logistic regression analysis was then performed to examine the relationship between the categorized emotional profiles and the occurrence of symptoms.

A significance level of 5% was maintained across all analyses. The data were stored in an anonymous base in Microsoft Excel 2013 (Microsoft Corporation, Redmond, WA, USA) and subsequently cleaned and analyzed using IBM SPSS software, Statistics for Windows (version 25, IBM Corp, Armonk, NY, USA), and R software (version 1.1.463, The R Foundation for Statistical Computing, Vienna, Austria).

### 2.8. Ethical Considerations

The present project was evaluated by the Clinical Research Ethics Committee of the Hospital Clínico San Carlos of Madrid, and a favorable report was obtained (C.I. 20/685-E). Likewise, a request for permission to use, process, exploit, and disseminate the data and information for the development of this research study was submitted to the Spanish Renal Foundation, receiving permission from the latter to carry this out. Before being included in this study, the patients received an information sheet explaining its purpose. Those who voluntarily wished to participate signed an informed consent form for the subsequent data collection. All participants gave their informed consent for this research to be carried out in accordance with the Declaration of Helsinki.

## 3. Results

**Figure 1:**
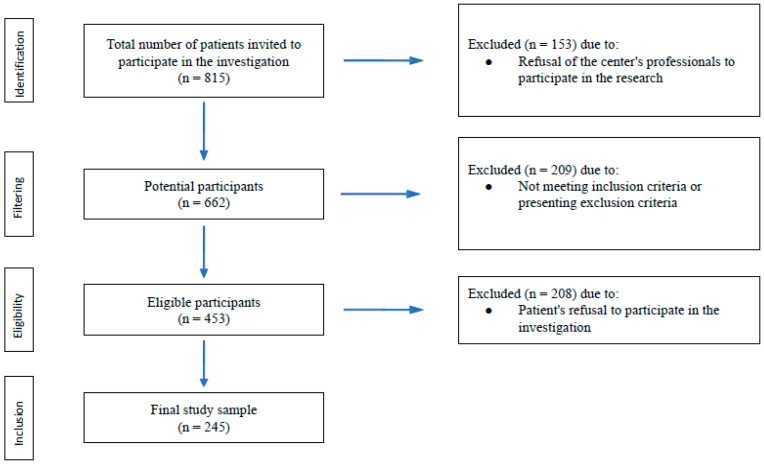
STROBE flowchart for the selection of participants.

The final study sample consisted of 245 patients from seven different centers of the Spanish Renal Foundation in the Community of Madrid (Figure 1).

The etiology of renal disease varied, with 22.9% cases of unknown origin (n = 53), followed by type II diabetes (20.3%; n = 47) and glomerulonephritis (11.3%; n = 26). The population analyzed was predominantly male (65.7%; n = 161), with a mean age of 63.52 years (SD 14.99) and a prolonged dialysis treatment duration of 81.44 months (SD 96.62). The clinical variables indicated that the patients received an adequate dialysis dose (mean Kt = 52.15), with interdialytic weight gains within the typical ranges (mean = 1.98 kg) and high Charlson Comorbidity Indexes (mean CCI = 7.68), reflecting a population with complex health needs.

Regarding the clinical variables, it should be noted that patients treated in different centers showed a similar profile, with statistically significant differences found only in the dialysis durations (*p* = 0.012) and average interdialytic weight gain (*p* = 0.035). The overall values obtained in the analysis of the clinical and sociodemographic variables distributed according to their center of origin can be found in Table 1.

**Figure 2:**
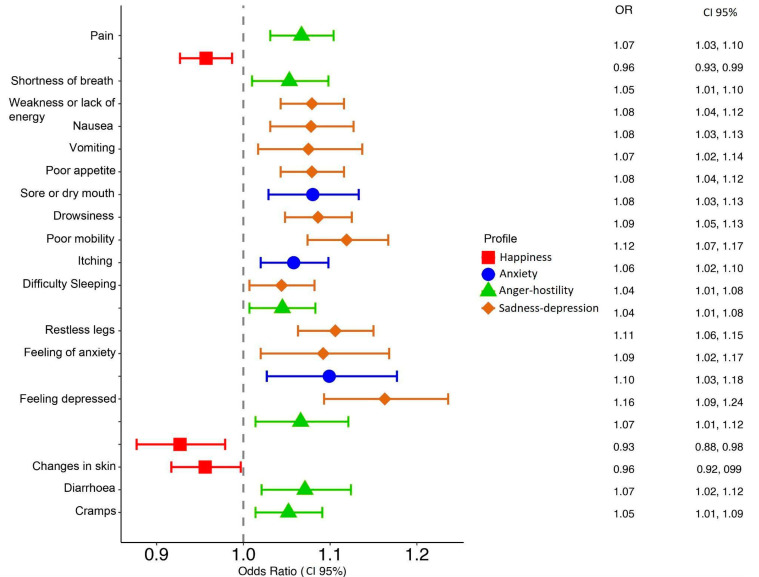
Relationship between the different emotional profiles and symptomatology. Note: Dotted line indicates an increase in risk (>1.00) or a decrease in risk (<1.00) in the exposed group.

Multivariate logistic regression, adjusted for the baseline covariates (age, sex, dialysis duration, final average Kt, and interdialytic weight gain), revealed significant associations between the emotional profiles and symptomatology. For each symptom, the relationship modeling it with the emotional profiles was obtained for patients of the same age, sex, time on dialysis, average final Kt, and interdialytic weight gain. Figure 2 illustrates the relationship between each emotional profile and the likelihood of experiencing specific symptoms. In general, as can be seen in Figure 2, negative emotional profiles (anxiety, anger–hostility, and sadness–depression) increase the risk of presenting symptoms, whereas the only favorable emotional profile (happiness) acts as a protective factor, reducing the appearance of some symptoms.

Table 2 details the odds ratios for each symptom in relation to the emotional profiles. Notably, patients with a sadness–depression profile had a higher risk of experiencing negative symptomatology than other profiles, with 10 related symptoms. This relationship affects both their physical and psychological symptomatology. In contrast, the happiness profile showed a protective effect against psychological symptoms and pain.

**Figure 3:**
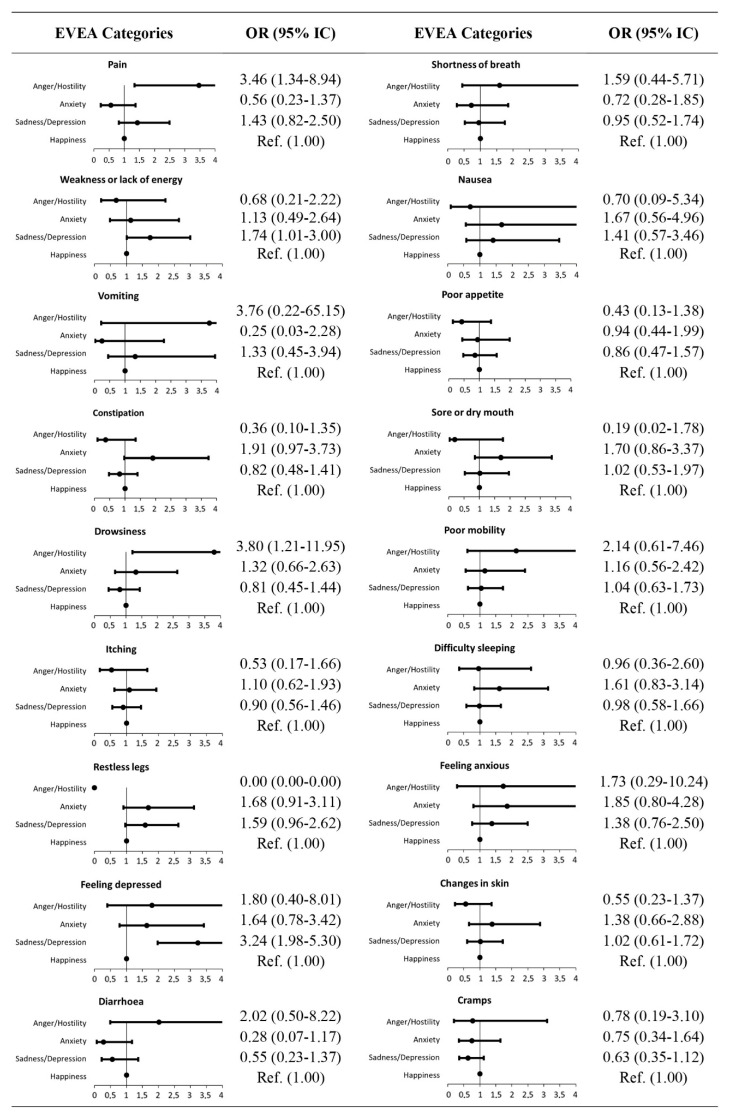
Forest plots of the Mood Evaluation Scale (EVEA) categories (symptoms according to EVEA categories vs. happiness).

Finally, a logistic regression analysis was performed considering, for each subject, the EVEA category in which they scored the highest in relation to the symptoms they experienced. The results, presented in Figure 3, reveal two statistically significant associations. On the one hand, the sadness–depression profile is related to a higher probability of experiencing weakness or a lack of energy (OR = 1.741; CI 95% 1.01–3.00) and feeling depressed (OR = 3.236; CI 95% 1.98–5.30). On the other hand, the anger–hostility profile shows a significant association with pain (OR = 3.463; CI 95% 1.34–8.94) and difficulty sleeping (OR = 3.796: CI 95% 1.21–11.95), indicating that the presence of this emotional state substantially increases the odds of developing these symptoms.

## 4. Discussion

The main objective of this study was to analyze the relationship between the emotional profiles of patients undergoing chronic HD and the symptoms they developed. These findings highlight the potential for early identification and targeted interventions aimed at managing emotional profiles that predispose patients to specific symptoms. In particular, the higher odds of experiencing fatigue and depressive feelings among patients with a sadness–depression profile suggest the need for emotional assessments and psychological support to mitigate these effects. Likewise, the association of the anger–hostility profile with pain and sleep disturbances underlines the importance of personalized approaches to symptom management. Addressing emotional profiles through psychological counseling and therapeutic communication could reduce the burden of symptoms in HD patients, supporting a comprehensive care model for CKD patients.

Regarding the happiness emotional profile, the results confirm its protective role against the development of limiting symptoms such as pain, depression, and skin changes. Satici and Uysal [26] noted that happiness is a predictor of psychological well-being and health perception, contributing to a reduced symptom burden. Positive emotions have been shown to exert a protective effect against negative emotions [27,28], a phenomenon supported by our findings, where patients with a happiness profile exhibited a decreased risk of feeling depressed. This aligns with evidence that positive emotions are associated with lower pain levels [29].

In contrast, negative emotions are associated with higher scores for this symptom [30]. Anxiety was significantly associated with somatic complaints such as itching. The literature supports the link between negative emotions and somatic symptoms like pruritus [31]. Additionally, the anxiety profile predicts a high frequency of somatic complaints, which can intensify and worsen the course and evolution of the disease, increasing morbidity [1,6]. Anxiety and depression often coexist, and both are directly related to an increased risk of hospitalization and longer durations of hospitalization [1,13]. Our findings in the sadness–depression profile are consistent with existing research demonstrating that depressive emotions in CKD patients are linked to physical decline, sleep disturbances, and impaired mobility [4,17,32,33,34,35,36]. Health problems can also elevate anxiety levels, establishing a bidirectional relationship between disease severity and emotional state [30]. The anger–hostility profile and the sadness–depression profile were linked to an increased risk of pain, shortness of breath, difficulty sleeping, feeling depressed, gastrointestinal symptoms, and cramps in a limiting way according to the results obtained in the multivariate analysis. Anger plays a recognized role in pain perception, particularly chronic pain [37,38,39,40,41]. The meta-analysis conducted by Adachi et al. [38] shows that anger is a predisposing and triggering factor for pain and even disability. Their study also refers to hostility, which, according to their conclusion, is not related to pain, in contrast to other authors such as McDermott et al. [40], who do find a direct relationship between a hostile emotional profile and the manifestation of this symptom. Our findings corroborate the hypothesis that hostility can exacerbate the expression of symptoms as found for other chronic conditions (such as irritable bowel disease) [31]. Notably, while some previous studies have identified a strong link between pain and depression [39,42], in our study, anger–hostility rather than sadness–depression was associated with pain. However, it has been seen in different investigations that anger acts as a mediator in the pain–depression relationship [37,40], so the results obtained here are not discordant. Finally, gastrointestinal symptoms, like nausea and vomiting, were also more frequent among patients with a sadness–depression profile, consistent with previous findings in elderly populations [43]. Non-adherence to the fluid restrictions required of these patients can seriously hamper their pulmonary function, which could explain the increased presence of respiratory distress and even insomnia, which is usually related, among other things, to excess interdialytic weight gain [44,45].

Managing the significant symptom burden experienced by people with chronic kidney disease undergoing HD requires a multidisciplinary approach, where nursing professionals play a pivotal role [14]. Emotions have a considerable impact on the manifestation and progression of their symptoms, directly influencing patients’ physical well-being. Given that nurses are the healthcare providers who spend the most time with patients during dialysis sessions, their continuous presence and close interaction position them as key agents in detecting subtle changes in mood or behavior that may indicate emotional distress [14]. This proximity makes nurses an essential part of the multidisciplinary team, acting as a vital link between patients and other healthcare specialists to ensure coordinated care and timely interventions. Through simple, routine measures—such as structured emotional assessments, direct communication, and careful observation—nurses can identify the early signs of emotional imbalance and intervene to prevent its potential impact on physical health [14,15]. This early detection is crucial, as the findings of this study suggest that patients’ emotional profiles may play a determining role in the onset and severity of their symptoms. By addressing emotional changes as they arise, nursing professionals can help mitigate complications, improve symptom management, and contribute to better clinical outcomes [14,15,46].

Beyond symptom monitoring, the nursing role encompasses ongoing emotional support, active listening, and the development of trust-based relationships that foster patient well-being [15]. While psychological care is essential, nurses’ ability to integrate emotional assessments into routine care allows for immediate, practical responses that enhance patients’ sense of security and emotional balance [14,16]. This proactive approach not only helps prevent symptom exacerbation but also strengthens patients’ adherence to treatment, promoting a greater sense of control over their health [16]. Moreover, having well-trained nursing staff with strong communication skills and emotional awareness is essential to creating a therapeutic environment during dialysis sessions. By facilitating health education, promoting emotional well-being, and working closely with the multidisciplinary team, nurses can contribute significantly to improving patients’ quality of life [14,15,46]. Their capacity to bridge physical and emotional care makes them indispensable in providing holistic, person-centered care for individuals living with chronic kidney disease. Ultimately, by recognizing and addressing the emotional needs of patients, nursing professionals act as fundamental agents in transforming the care experience. Their dedication not only alleviates suffering but also promotes dignity, resilience, and overall well-being, reinforcing the essential role of nursing in enhancing both the physical and emotional health outcomes in patients undergoing HD [14,15].

Among the limitations of this research are the convenience sampling and the measurement instruments used since both scales are subjective measures of the main study variables. This limits the external validity of the results obtained and requires future research with a larger sample that represents the study population better. Additionally, the cross-sectional design of this study precludes the ability to infer causality between the variables; longitudinal studies are therefore needed to explore the temporal dynamics and potential causal pathways involved in the relationships observed. Regarding the measurement instruments, it should be taken into account that there are multiple scales that can be used to measure symptoms and emotions, with different variables and scores, which makes it difficult to compare the results when the same instruments are not used to measure them, which again conditions the generalizability of the results obtained. Furthermore, this study did not account for several potential confounding variables that could influence the presence of symptoms and emotional profiles, such as levels of family or social support, exposure to recent stressful life events, or the presence of undiagnosed psychiatric conditions. These unmeasured factors may have biased the results and should be systematically assessed in future research to isolate the effects of the primary variables under study better. Finally, future investigations should explore the influence of additional demographic and clinical variables—such as sex, age, and the Charlson Comorbidity Index (CCI)—on both the presence of symptoms and emotional profiles using designs and methodologies that would allow for a more nuanced understanding of the complex interactions among these factors.

## 5. Conclusions

This study highlights the strong association between the emotional profiles and the symptom burden in CKD patients on HD. Negative emotions—such as anger, sadness, and anxiety—are linked to more severe symptoms, while happiness shows a protective effect, even mitigating the impact of coexisting negative states. These findings emphasize the importance of integrating emotional assessments into routine clinical care. Nurses, due to their close contact with patients, are ideally positioned to monitor emotional well-being and intervene early. Future research should focus on evaluating psychosocial interventions and longitudinal outcomes, while healthcare policies should promote emotional support as part of the standard nephrology care.

## Figures and Tables

**Table 1:** Descriptive analysis of clinical and sociodemographic variables by center of origin.

Variables ^1^	Total (n = 245)	Rey Juan Carlos University Hospital (n = 26)	Fundación Jiménez Díaz University Hospital (n = 26)	Villalba General University Hospital (n = 38)	Los Lauros Center (n = 22)	Getafe Center (n = 40)	Santa Engracia Center (n = 81)	Infanta Elena University Hospital (n = 12)	*p*-Value
Age (years)	63.52 [61.69–65.51]	64.81 [59.05–70.57]	65.15 [60.33–69.98]	62.11 [56.94–67.27]	65.09 [59.58–0.61]	58.65 [53.63–3.67]	65.17 [61.63–68.72]	63.92 [54.97–2.86]	0.421
Time on dialysis (months)	81.44 [69.13–93.76]	70.24 [47.13–93.35]	121.00 [86.50–155.50]	123.43 [80.01–166.81]	49.96 [19.04–80.87]	36.10 [18.59–53.61]	72.53 [51.54–93.53]	154.92 [79.96–229.87]	0.012 *
Average final Kt	52.15 [51.05–3.38]	47.99 [44.79–51.19]	53.7 [48.95–57.18]	49.13 [46.63–51.63]	54.96 [50.40–59.51]	56.08 [54.17–57.99]	52.29 [50.11–54.47]	49.56 [44.23–54.89]	0.763
Average interdialytic weight gain (Kg)	1.98 [1.87–2.05]	2.03 [1.67–2.39]	2.00 [1.75–2.25]	2.05 [1.85–2.25]	1.78 [1.35–2.22]	1.96 [1.72–2.19]	2.00 [1.86–2.14]	1.93 [1.47–2.38]	0.035 *
Charlson Comorbidity Index	7.68 [7.15–8.21]	8.80 [6.79–10.81]	8.08 [6.84–9.32]	7.45 [6.12–8.78]	6.38 [5.12–7.63]	6.0 [4.30–7.70]	7.89 [6.61–9.16]	8.38 [5.28–11.47]	0.192
Men	161 (65.7%)	17 (65.4%)	19 (73.1%)	20 (52.6%)	14 (63.6%)	26 (65.0%)	55 (67.9%)	10 (83.3%)	0.496
Women	84 (34.3%)	9 (34.6%)	7 (26.9%)	18 (47.4%)	8 (36.4%)	14 (35.0%)	26 (32.1%)	2 (16.7%)	
Permanent catheter	69 (28.2%)	7 (26.9%)	9 (34.6%)	19 (50.0%)	7 (31.8%)	9 (22.5%)	15 (18.5%)	3 (25.0%)	0.054
Arteriovenous fistula	176 (71.8%)	19 (73.1%)	17 (65.4%)	19 (50.0%)	15 (68.2%)	31 (77.5%)	66 (81.5%)	9 (75.0%)	
Type I diabetes	8 (3.5%)	0 (0.0%)	0 (0.0%)	4 (11.1%)	1 (5.6%)	3 (7.5%)	0 (0.0%)	0 (0.0%)	0.037 *
Type II diabetes	47 (20.3%)	7 (28.0%)	4 (16.0%)	5 (13.9%)	2 (11.1%)	10 (25.0%)	16 (20.5%)	3 (33.3%)	0.604
Cystic kidney disease, unspecified type	1 (0.4%)	0 (0.0%)	0 (0.0%)	0 (0.0%)	0 (0.0%)	0 (0.0%)	0 (0.0%)	1 (11.1%)	-
Hypertensive vascular kidney disease	24 (10.4%)	3 (12.0%)	1 (4.0%)	4 (11.1%)	4 (22.2%)	3 (7.5%)	9 (11.5%)	0 (0.0%)	0.493
Multisystemic diseases—other	7 (3.0%)	1 (4.0%)	2 (8.0%)	2 (5.6%)	0 (0.0%)	0 (0.0%)	2 (2.6%)	0 (0.0%)	0.529
Glomerulonephritis	26 (11.3%)	0 (0.0%)	5 (20.0%)	3 (8.3%)	0 (0.0%)	8 (20.0%)	9 (11.5%)	1 (11.1%)	0.097
Chronic kidney failure (unknown etiology)	53 (22.9%)	1 (4.0%)	7 (28.0%)	4 (11.1%)	4 (22.2%)	6 (15.0%)	29 (37.2%)	2 (22.2%)	0.005 **
Tubular necrosis, cortical necrosis	1 (0.4%)	1 (4.0%)	0 (0.0%)	0 (0.0%)	0 (0.0%)	0 (0.0%)	0 (0.0%)	0 (0.0%)	0.219
Nephroangiosclerosis	1 (0.4%)	0 (0.0%)	1 (4.0%)	0 (0.0%)	0 (0.0%)	0 (0.0%)	0 (0.0%)	0 (0.0%)	-
Hereditary nephropathy	2 (0.9%)	0 (0.0%)	0 (0.0%)	2 (5.6%)	0 (0.0%)	0 (0.0%)	0 (0.0%)	0 (0.0%)	-
Membranous nephropathy	1 (0.4%)	0 (0.0%)	0 (0.0%)	1 (2.8%)	0 (0.0%)	0 (0.0%)	0 (0.0%)	0 (0.0%)	-
IgA nephropathy	7 (3.0%)	0 (0.0%)	0 (0.0%)	1 (2.8%)	2 (11.1%)	1 (2.5%)	3 (3.8%)	0 (0.0%)	-
Nephropathy due to a specific drug	1 (0.4%)	0 (0.0%)	0 (0.0%)	1 (2.8%)	0 (0.0%)	0 (0.0%)	0 (0.0%)	0 (0.0%)	0.489
Nephropathy secondary to polycystic kidney disease	1 (0.4%)	0 (0.0%)	0 (0.0%)	1 (2.8%)	0 (0.0%)	0 (0.0%)	0 (0.0%)	0 (0.0%)	0.489
Other classifiable vascular kidney diseases	3 (1.3%)	0 (0.0%)	0 (0.0%)	2 (5.6%)	1 (5.6%)	0 (0.0%)	0 (0.0%)	0 (0.0%)	0.126
Other specific kidney disorders	6 (2.6%)	0 (0.0%)	2 (8.0%)	1 (2.8%)	0 (0.0%)	1 (2.5%)	2 (2.6%)	0 (0.0%)	0.639
Traumatic or surgical kidney loss	1 (0.4%)	0 (0.0%)	0 (0.0%)	1 (2.8%)	0 (0.0%)	0 (0.0%)	0 (0.0%)	0 (0.0%)	0.489
Pyelonephritis/interstitial nephritis	15 (6.5%)	2 (8.0%)	0 (0.0%)	1 (2.8%)	3 (16.7%)	3 (7.5%)	5 (6.4%)	1 (11.1%)	0.412
Polycystic kidneys	15 (6.5%)	2 (8.0%)	2 (8.0%)	1 (2.8%)	1 (5.6%)	5 (12.5%)	3 (3.8%)	1 (11.1%)	0.405
Renal tumors	1 (0.4%)	0 (0.0%)	1 (4.0%)	0 (0.0%)	0 (0.0%)	0 (0.0%)	0 (0.0%)	0 (0.0%)	0.219

^1^ Qualitative variables as numbers and percentages, n (%); quantitative variables as mean and CI 95%, mean [CI 95%]. * *p*-value < 0.05, ** *p*-value < 0.01. The emotional profile analysis showed that most patients scored high on the happiness profile, with a mean score of 23.11 (SD = 10.97), while negative profiles scored lower, such as the anger–hostility profile (mean = 7.16; SD = 8.77). In particular, the happiness profile scores exceeded 20 points in more than 50% of the participants and surpassed 30 points in 25%. In contrast, the negative emotional profiles (sadness–depression, anger–hostility, and anxiety) scored below 6 points in 50% of the sample.

**Table 2:** Odds ratio relationship between the different emotional profiles and symptomatology.

Variables ^1^	Sadness–Depression Profile	Anger–Hostility Profile	Anxiety Profile	Happiness Profile
Pain	*p* > 0.05	1.07 [1.03–1.10]	*p* > 0.05	0.96 [0.93–0.99]
Shortness of breath	*p* > 0.05	1.05 [1.01–1.10]	*p* > 0.05	*p* > 0.05
Weakness or a lack of energy	1.08 [1.04–1.12]	*p* > 0.05	*p* > 0.05	*p* > 0.05
Nausea	1.08 [1.03–1.13]	*p* > 0.05	*p* > 0.05	*p* > 0.05
Vomiting	1.08 [1.02–1.14]	*p* > 0.05	*p* > 0.05	*p* > 0.05
Poor appetite	1.08 [1.04–1.12]	*p* > 0.05	*p* > 0.05	*p* > 0.05
Constipation	*p* > 0.05	*p* > 0.05	*p* > 0.05	*p* > 0.05
Sore or dry mouth	*p* > 0.05	*p* > 0.05	1.08 [1.03–1.13]	*p* > 0.05
Drowsiness	1.09 [1.05–1.13]	*p* > 0.05	*p* > 0.05	*p* > 0.05
Poor mobility	1.12 [1.07–1.17]	*p* > 0.05	*p* > 0.05	*p* > 0.05
Itching	*p* > 0.05	*p* > 0.05	1.06 [1.02–1.10]	*p* > 0.05
Difficulty sleeping	1.04 [1.01–1.08]	1.05 [1.01–1.08]	*p* > 0.05	*p* > 0.05
Restless legs	1.11 [1.06–1.15]	*p* > 0.05	*p* > 0.05	*p* > 0.05
Feeling anxious	1.09 [1.02–1.17]	*p* > 0.05	1.10 [1.03–1.18]	*p* > 0.05
Feeling depressed	1.16 [1.09–1.24]	1.07 [1.01–1.12]	*p* > 0.05	0.93 [0.88–0.99]
Changes in skin	*p* > 0.05	confounding variable	*p* > 0.05	0.96 [0.92–0.99]
Diarrhea	*p* > 0.05	1.07 [1.02–1.12]	*p* > 0.05	*p* > 0.05
Cramps	*p* > 0.05	1.05 [1.01–1.09]	*p* > 0.05	*p* > 0.05

^1^ Odds ratios and 95% CIs for statistically significant differences/*p* > 0.05 used for non-statistically significant differences.

## Data Availability

The data supporting this study’s findings are available from the corresponding author upon reasonable request.

## Public Involvement Statement

There was no public involvement in any aspect of this research.

## Guidelines and Standards Statement

This manuscript was drafted against the STROBE (Strengthening the Reporting of Observational studies in Epidemiology) statement for observational studies.
